# Protein Subdomain Enrichment of *NUP155* Variants Identify a Novel Predicted Pathogenic Hotspot

**DOI:** 10.3389/fcvm.2020.00008

**Published:** 2020-02-07

**Authors:** Riley J. Leonard, Claudia C. Preston, Melanie E. Gucwa, Yohannes Afeworki, Arielle S. Selya, Randolph S. Faustino

**Affiliations:** ^1^Genetics and Genomics Group, Sanford Research, Sioux Falls, SD, United States; ^2^Department of Biology, College of St. Benedict/St. John's University, Collegeville, MN, United States; ^3^Department of Biology, Carthage College, Kenosha, WI, United States; ^4^Functional Genomics & Bioinformatics Core Facility, Sanford Research, Sioux Falls, SD, United States; ^5^Behavioral Sciences Group, Sanford Research, Sioux Falls, SD, United States; ^6^Department of Pediatrics, Sanford School of Medicine of the University of South Dakota, Sioux Falls, SD, United States

**Keywords:** single nucleotide variants (SNV), nucleoporins, network biology and protein-protein interactions, atrial fibrillation (AF), nuclear envelope (NE)

## Abstract

Functional variants in nuclear envelope genes are implicated as underlying causes of cardiopathology. To examine the potential association of single nucleotide variants of nucleoporin genes with cardiac disease, we employed a prognostic scoring approach to investigate variants of *NUP155*, a nucleoporin gene clinically linked with atrial fibrillation. Here we implemented bioinformatic profiling and predictive scoring, based on the gnomAD, National Heart Lung and Blood Institute-Exome Sequencing Project (NHLBI-ESP) Exome Variant Server, and dbNSFP databases to identify rare single nucleotide variants (SNVs) of *NUP155* potentially associated with cardiopathology. This predictive scoring revealed 24 SNVs of *NUP155* as potentially cardiopathogenic variants located primarily in the N-terminal crescent-shaped domain of NUP155. In addition, a predicted NUP155 R672G variant prioritized in our study was mapped to a region within the alpha helical stack of the crescent domain of NUP155. Bioinformatic analysis of inferred protein-protein interactions of NUP155 revealed over representation of top functions related to molecular transport, RNA trafficking, and RNA post-transcriptional modification. Topology analysis revealed prioritized hubs critical for maintaining network integrity and informational flow that included *FN1, SIRT7*, and *CUL7* with nodal enrichment of RNA helicases in the topmost enriched subnetwork. Furthermore, integration of the top 5 subnetworks to capture network topology of an expanded framework revealed that *FN1* maintained its hub status, with elevation of *EED, CUL3*, and *EFTUD2*. This is the first study to report novel discovery of a NUP155 subdomain hotspot that enriches for allelic variants of NUP155 predicted to be clinically damaging, and supports a role for RNA metabolism in cardiac disease and development.

## Introduction

Atrial fibrillation (AF) is the most prevalent arrhythmia reported in the clinic, and as the population ages, a significant increase in the global burden of this disease is expected within the next 50 years (1, 2). AF is marked by a poor ability to function under exertion and an increased prevalence of stroke and heart failure (3). In addition to this diminished quality of life, undiagnosed AF cases paired with an incomplete knowledge of its molecular basis confounds mitigation of this burgeoning epidemic. Better understanding of AF etiology is thus mandatory for developing advanced strategies to address this disease (4).

Nuclear envelope genes have emerged as a novel pool of candidates that impact normal cardiac function (5, 6). Indeed, reported gene disruptions in all major components of the nuclear envelope, which include the nuclear lamina, the linker of nucleus and cytoskeleton complex (LINC) and the multimeric nuclear pore complex (NPC) have been shown to facilitate or associate with cardiopathogenesis (7, 8). Of these, the nuclear lamina and LINC complex have been better characterized with respect to their role in cardiopathology, with recent studies beginning to recognize potential functional roles for the NPC and its individual nucleoporins (nups) in cardiac disease. Indeed, earlier studies had identified a NUP155 R391H variant as an inherited underlying cause of atrial fibrillation and sudden pediatric cardiac death in multiple generations of a South American family, while independent work revealed a NUP155 L503F variant associated with sudden cardiac death in a rural Chinese population (9, 10). Further evidence for the role of the nuclear envelope in these *NUP155* clinical cases is supported by our work as well as others (11–13), but whether or not other NUP155 variants may be pathogenic remains unknown. As these previously mentioned NUP155 missense mutations as well as dysregulated expression of other discrete nups have been associated with a variety of clinical cardiopathologies (5, 10, 14), this study was carried out to investigate the prevalence of reported NUP155 variants with potential cardiopathogenicity. To this end, we canvassed variants within the NHLBI Exome Sequencing Project along with data from gnomAD and dbNSFP databases to enhance prioritization of variants potentially implicated in cardiovascular disorders (15, 16).

## Materials and Methods

### Databases and Data Collection

Three databases were accessed covering *NUP155* gene variants and data was downloaded for further analysis. The National Heart Lung Blood Institute-Exome Sequencing Project (NHLBI-ESP) Exome Variant Server (last accessed on 12/30/19), the Genome Aggregation Database (gnomAD) v2.1.1 (accessed on 12/30/19), and the dbNSFP database v4.0a (accessed on 12/20/2019) (17). NHLBI-ESP Exome Variant Server (EVS) is comprised of 6503 samples from European- Americans (*n* = 4,300) and African-Americans (*n* = 2,203) represented in a variety of established studies investigating cardiovascular disease within well-characterized populations (controls, extremes of specific clinical traits, and specific cardiovascular and lung diseases). The gnomAD database is an online repository containing 125,748 exome sequences and 15,708 whole-genome sequences from unrelated individuals and subsumes exome data from the original 60,706 individuals within the ExAC dataset (18). The dbNSFP database was developed for functional prediction and annotation of all potential non-synonymous single-nucleotide variants (nsSNVs) and splice site variants (ssSNVs) in the human genome. It comprises a total of 84,013,490 nsSNVs and ssSNVs. All the *NUP155* gene variants were downloaded from each database and a Venn diagram analysis was conducted using the online tool found at http://bioinformatics.psb.ugent.be/webtools/Venn/.

### Bioinformatic and Biostatistical Analyses: Variant Prioritization

Variant prioritization was determined using the following metrics modeled after the approach used by Giudicessi and colleagues: Genomic Evolutionary Rate Profiling (GERP); PhastCons; Grantham Score; PolyPhen-2; Protein Variation Effect Analyzer (PROVEAN); and Sorting Intolerant From Tolerant (SIFT) (19–23). GERP is a nucleotide conservation score that estimates evolutionary rates for nucleotides in a multi-species alignment, and compares these inferred rates with a phylogenetic tree describing neutral substitution rates relating the species under consideration (20). Scores range from −12.3 to 6.17, with 6.17 being the most conserved. PhastCons conservation score describes the degree of sequence conservation among 17 vertebrate species, where scores fall within a range between 0 and 1.

In addition to nucleotide conservation scoring, amino acid change predictions were considered as well. Grantham scoring ranges from 5 to 215 and predicts evolutionary distances between amino acid changes. Scores above 125 are considered “probably-damaging.” PolyPhen-2 scores predict possible effects of amino acid substitutions on overall protein structure and function. Scores range from 0.0 (tolerated) to 1.0 (deleterious), where >0.85 is more confidently predicted to be “probably-damaging.” PROVEAN is a software tool that predicts whether an amino acid change will have an effect on biological function of the protein. PROVEAN scores below −2.5 is considered “deleterious,” while those greater than that threshold is considered “neutral.” SIFT scoring predicts impact of amino acid substitution on protein function, and ranges from 0 to 1, with scores <0.05 considered “deleterious.”

To filter variants, scores beyond deleterious thresholds for each metric were used to prioritize variants. In this manner, all variants that met or exceeded threshold values for all metrics were prioritized for consideration as a predicted pathogenic variant. For example, we started with the amino acid change prediction scores, PolyPhen2 class prediction, and focused on the extreme class of “probably damaging” alone and then moved to Grantham, PROVEAN and SIFT scoring and ended with the GERP and PhastCons conservation scores. For further refinement, we implemented a minor allele frequency (MAF) threshold filtering step based on gnomAD derived MAF for confirmed pathogenic variants of *SCN5A*, a known AF gene (24). This resulted in our final prioritized list of *NUP155* rare variants.

### Variant Hotspot Analysis

Nonrandom clustering was examined using the statistical procedure and R code described in Ye et al. (25), which identifies clusters empirically without specifying the number of mutations or the cluster length, and included the Benjamini-Hochberg correction for multiple comparisons. Analysis of a bootstrapped dataset (*n* = 1,000) generated from the prioritized list of potentially pathogenic NUP155 variants was performed to generate a list of statistically significant clusters of varying size, along with the size and location (start and end positions) of each cluster. The number of significant clusters at each position was summed and displayed as a heatmap adjacent to the mapped position of each NUP155 variant identified in this study to visualize hotspots of variant clusters.

### 3D Structural Modeling

PyMOL version 2.3 (https://pymol.org/2/, Schrödinger, Cambridge, MA) was used for 3D rendering and visualization of NUP155, Nup157, and Nup170. To visualize the protein conformation of NUP155 for the present study, the RCSB PDB identifiers 5IJO.A (Entity ID: 1), 5IJN.E, 4MHC, and 5HAX were used. (26) NUP155 protein (Chain A) was prioritized for analysis.

### Network Cartography and Parameter Analysis

To investigate the potential network of NUP155-related proteins, a list of inferred human NUP155 protein-protein interactions was analyzed as follows. Potential NUP155-interacting protein identifiers were mined from the GeneCards database, then submitted to Ingenuity Pathway Analysis (IPA, Qiagen, Germantown, MD) to map inferred network pathways. Analysis settings for IPA were set to report direct and indirect relationships and filtering criteria were set to include only experimentally observed relationships. A total of 21 subnetworks were identified, each one constructed of 35 nodes, and the top 5 subnetworks were assembled into one inclusive network using the “Merge Networks” function within IPA. Edges within this collective network indicate functional interactions curated within the Ingenuity Knowledge Base. These relationship data were collated and exported in.xls format using the “Export Data **→** Export **→** All Relationships” feature within IPA, and served as an input file for further network analysis in Cytoscape (https://cytoscape.org/), as previously performed (12). Briefly, the “Network Analyzer” plugin from Cytoscape was used to quantify network topology parameters that informed network metrics scores including neighborhood connectivity, betweenness and closeness centrality scores.

## Results

### Prediction of Potentially Damaging Missense Variants of *NUP155*

**Figure 1:**
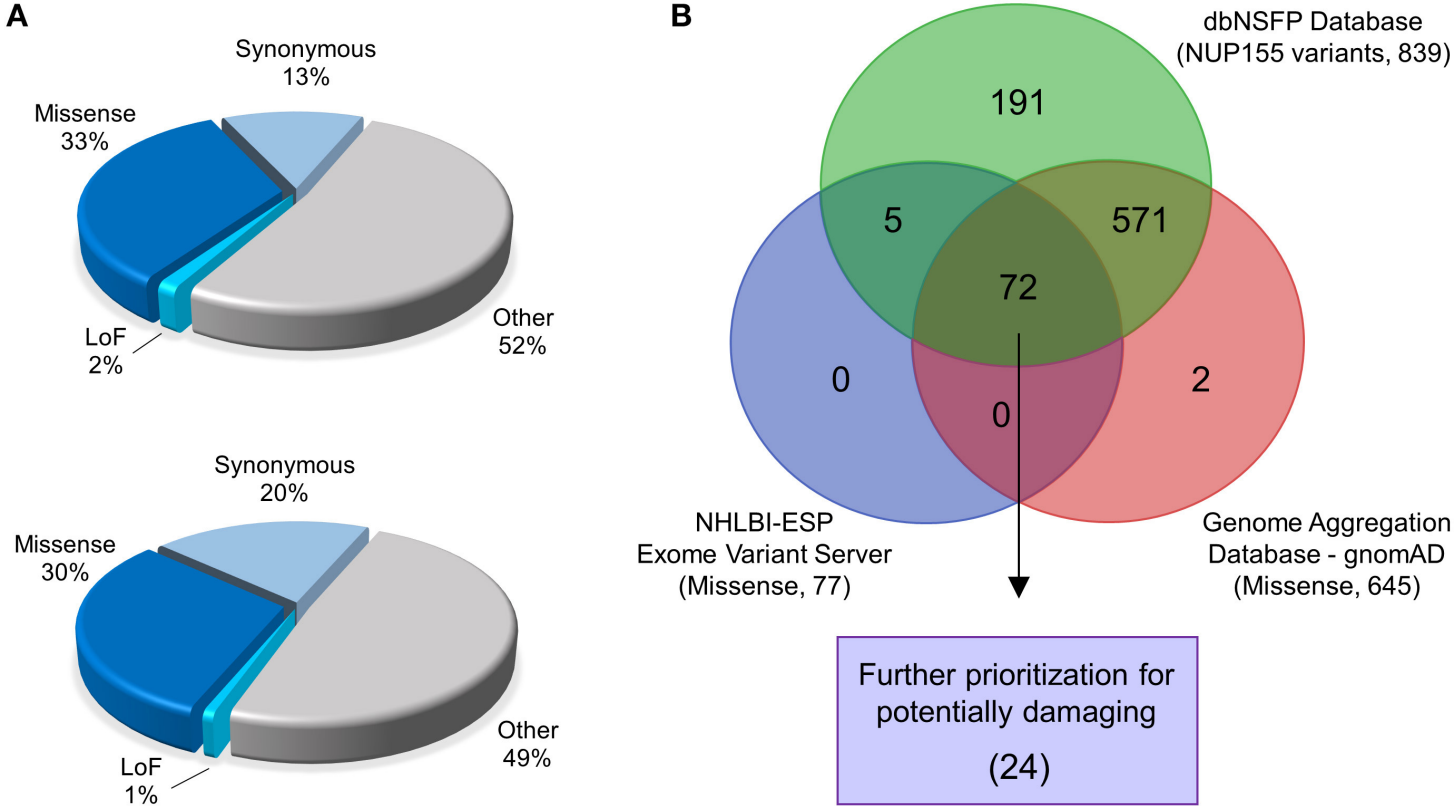
Identification of prioritized variants of *NUP155*. **(A)** The gnomAD (top pie chart) and NHLBI-EVS (bottom pie chart) databases were used to identify potential pathogenic nup variants in the context of cardiovascular disease cohorts. Schematic pie charts represent synonymous, missense, loss of function (LoF) and other (untranslated regions, splice region, and intronic sequences) mutations in each database, respectively. **(B)** Missense nup variants in the gnomAD database were compared to those in the NHLBI-EVS and the dbNSFP datasets to identify variant overlap. From a total of 72 variants determined from all three databases, 24 variants were prioritized as probably damaging.

Analysis of *NUP155* single nucleotide variants (SNVs) reported in gnomAD returned a total of 2176 variants. These were distributed among loss-of-function (that includes annotations of “stop gained”, “splice donor”, and “frameshift”) (30), missense (724), synonymous (290), and other (1132) categories (Figure 1A). Variants that did not pass gnomAD quality control were excluded. Sub-categories within “other” included variants located in 5′ and 3′ untranslated regions (UTRs), splice region, and intronic sequences. Start/stop loss insertion/deletions (12), duplicates (55), and those without unique rsIDs (12) were filtered out of the 724 protein coding variants found in gnomAD for a total of 645 for further analysis.

**Figure 2:**
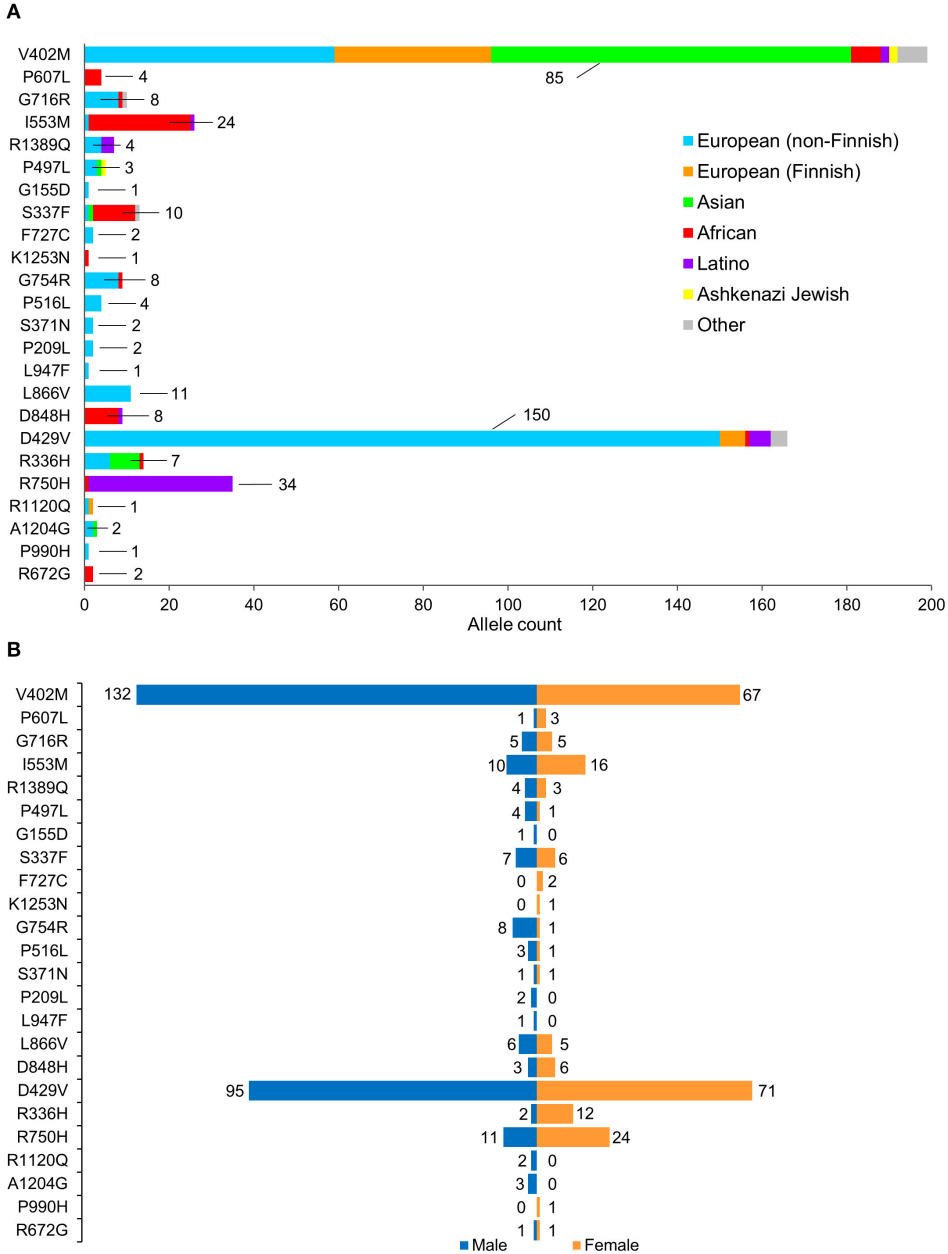
Population characteristics of cohorts associated with prioritized *NUP155* variants. **(A)** Occurrence of the *NUP155* prioritized variants cohort in European (non-Finnish), European (Finnish), Asian (including East Asians), African, Latino and Ashkenazi Jewish populations based on gnomAD database information. “Other” ethnicity includes individuals that did not classify into given gnomAD designations. Highlighted here are the numbers of the most overrepresented ethnic population for each variant. Total allele counts based on population characteristics is shown in Supplemental Table 2. **(B)** Breakdown of the *NUP155* prioritized variants occurrence according to sex, where males are shown in blue and females in orange.

A total of 257 NUP155 variants were identified in the NHLBI-EVS dataset, which included 2 loss-of-function mutations, 77 missense, 51 synonymous and 126 referred to as “Other” (Figure 1A). Venn diagram analysis revealed a total of 72 protein coding NUP155 variants common to all three databases. Variant prioritization was determined using four amino acid change prediction scores and two variant conservation scores. Predictive scoring for all 72 variants is shown in Supplemental Table 1. After prioritization, 24 variants were predicted as the most potentially damaging (Figure 1B and Table 1). When filtering according to MAF thresholds defined by pathogenic AF-associated *SCN5A* variants, 23 out of 24 *NUP155* variants possessed a MAF below that of the rarer S216L *SCN5A* variant (MAF = 6.5 × 10^−4^), while the remaining V402M *NUP155* variant possessed a MAF of 7.0 × 10^−4^ below that of the less rare, but still pathogenic F2004L *SCN5A* variant (MAF = 1.9 × 10^−3^) (Table 1). Population characteristic distribution (ethnicity and sex data) was extracted from gnomAD that showed all 24 variants inherited in heterozygous form. Ethnicity distribution revealed different diversity patterns for each variant (Figures 2A,B). Of interest, 13 out of the 24 variants were overrepresented in European (non-Finnish) individuals. Moreover, V402M showed the most diverse pattern distribution based on ethnicity, with the majority of allelic changes reported in males (Figure 2A). Total allele count population characteristics for all 24 prioritized variants are provided in Supplemental Table 2.

**Table 1:** Novel potentially damaging *NUP155* variants.

**Variant rs_IDs**	**Protein Change**	**PolyPhen2 (Class:Score)**	**Grantham Score**	**Provean Score**	**SIFT Score**	**PhastCons score**	**GERP score**	**Allele frequency (%)**
rs148814027	V402M	Probably-damaging:0.971	21	−3.24*	0.004*	1	5.68	0.000704
rs371676330	P607L	Probably-damaging:0.975	98	−2.16	0.008*	0.985	5.07	0.000016
rs200783324	G716R	Probably-damaging:0.984	125*	−1.65	0.002*	0.998	5.78	0.000035
rs373000659	I553M	Probably-damaging:0.985	10	−2.83*	0.013*	0.988	−1.05	0.000092
rs202194194	R1389Q	Probably-damaging:0.987	43	−5.81*	0.141	1	5.05	0.000025
rs148457088	P497L	Probably-damaging:0.989	98	−4.15*	0.112	1	5.41	0.000020
rs376772699	G155D	Probably-damaging:0.993	94	−4.91*	0.005*	0.993	4.85	0.000004
rs142350078	S337F	Probably-damaging:0.994	155*	−2.55*	0.061	1	5.46	0.000046
rs368777239	F727C	Probably-damaging:0.995	205*	−1.91	0.176	0.999	5.78	0.000008
rs151163391	K1253N	Probably-damaging:0.996	94	−1.46	0.496	0.971	0.43	0.000004
rs145147317	G754R	Probably-damaging:0.997	125*	−6.36*	0.002*	1	4.49	0.000032
rs375239602	P516L	Probably-damaging:0.997	98	−1.27	0.253	1	5.55	0.000014
rs143375056	S371N	Probably-damaging:0.998	46	−5.97*	0.007*	1	3.64	0.000004
rs370781964	P209L	Probably-damaging:0.998	98	−5.97*	0.007*	1	5.83	0.000008
rs149244067	L947F	Probably-damaging:0.999	22	−2.61*	0*	0.995	4.59	0.000004
rs145975462	L866V	Probably-damaging:0.999	32	−6.5*	0.002*	1	6.16	0.000039
rs142961329	D848H	Probably-damaging:0.999	81	−6.27*	0.001*	1	5.85	0.000032
rs141688173	D429V	Probably-damaging:0.999	152*	−3.08*	0.009*	1	5.07	0.000591
rs376696300	R336H	Probably-damaging:1.0	29	−1.99	0.024*	1	5.46	0.000056
rs202058711	R750H	Probably-damaging:1.0	29	−6.5*	0*	1	5.59	0.000139
rs373119361	R1120Q	Probably-damaging:1.0	43	−4.43*	0.001*	1	5.44	0.000008
rs145640004	A1204G	Probably-damaging:1.0	60	−3.59*	0*	1	5.11	0.000012
rs376271013	P990H	Probably-damaging:1.0	77	−1.27	0.017*	1	4.8	0.000004
rs373376199	R672G	Probably-damaging:1.0	125*	−6.5*	0.024*	1	5.16	0.000007

*Variants for NUP155 common to the dbNSFP, NHLBI-EVS and gnomAD datasets that were prioritized as probably damaging variants (see methodology) and are listed here with their respective predictive scores, rs IDs and allele frequencies (based on gnomAD database). Genomic Evolutionary Rate Profiling (GERP) and PhastCons are evolutionary conservation scores; the Grantham, PolyPhen-2, Protein Variation Effect Analyzer (PROVEAN) and Sorting Intolerant From Tolerant (SIFT) scores are metrics that predict potential radical amino acid substitutions. The amino acid substitution prediction scores that are considered deleterious are highlighted with a star*.

### Prioritized Variants Cluster Within a Discrete NUP155 Subdomain

**Figure 3:**
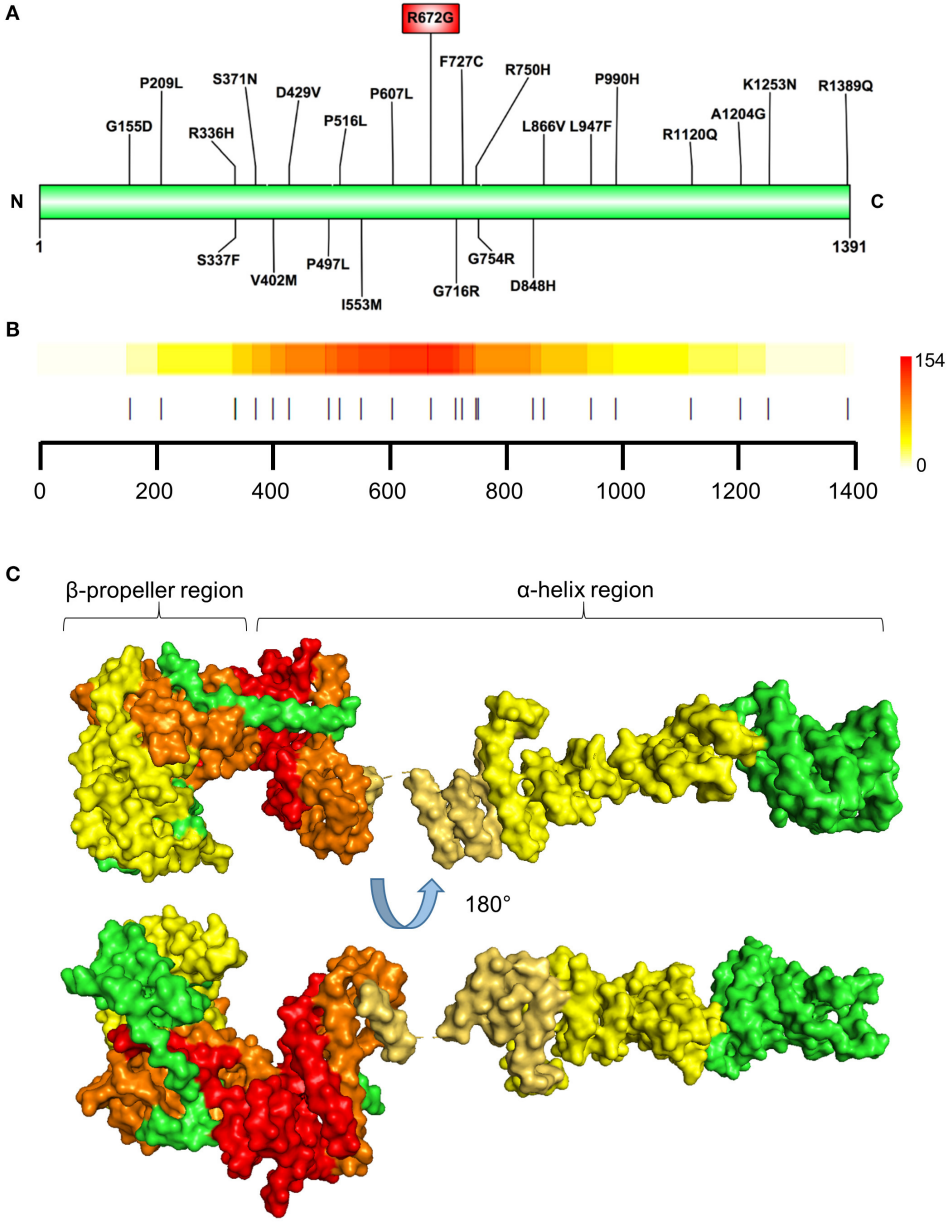
Identification of prioritized variant hostpot. **(A)** Schematic illustration showing locations of the *NUP155* prioritized variants. Highest prioritized variant identified in the present analysis (R672G) indicated in the red rectangle. **(B)** Variant cluster analysis defines a hotspot within the NUP155 protein. Localization of all variants predicted to be highly pathogenic depicted. Colorscale to the right indicates low-to-high range clustering enrichment scores (0–154). Red indicates high clustering regions. Horizontal axis provides amino acid position as shown in **(A)** with each prioritized variant indicated by a vertical line. **(C)** Surface rendering of a predicted 3D model of NUP155 reveals enrichment of the cardiopathogenic cluster in the crescent-shaped N-terminal domain of the protein. Top and bottom illustrations show alternate rotated views to highlight distribution of low and high clustered regions. Green, NUP155; yellow, low clustering; orange, midrange; red, high.

Prioritized variants in the NUP155 protein were mapped to the linear amino acid representation of NUP155 and clustered within a specific N-terminal domain of NUP155 (Figures 3A,B). Distribution of these prioritized variants in the context of NUP155 secondary and tertiary structure revealed that the majority of the variants of interest (*p* < 0.05) are enriched within a crescent-shaped domain of NUP155 (Figure 3C).

**Figure 4:**
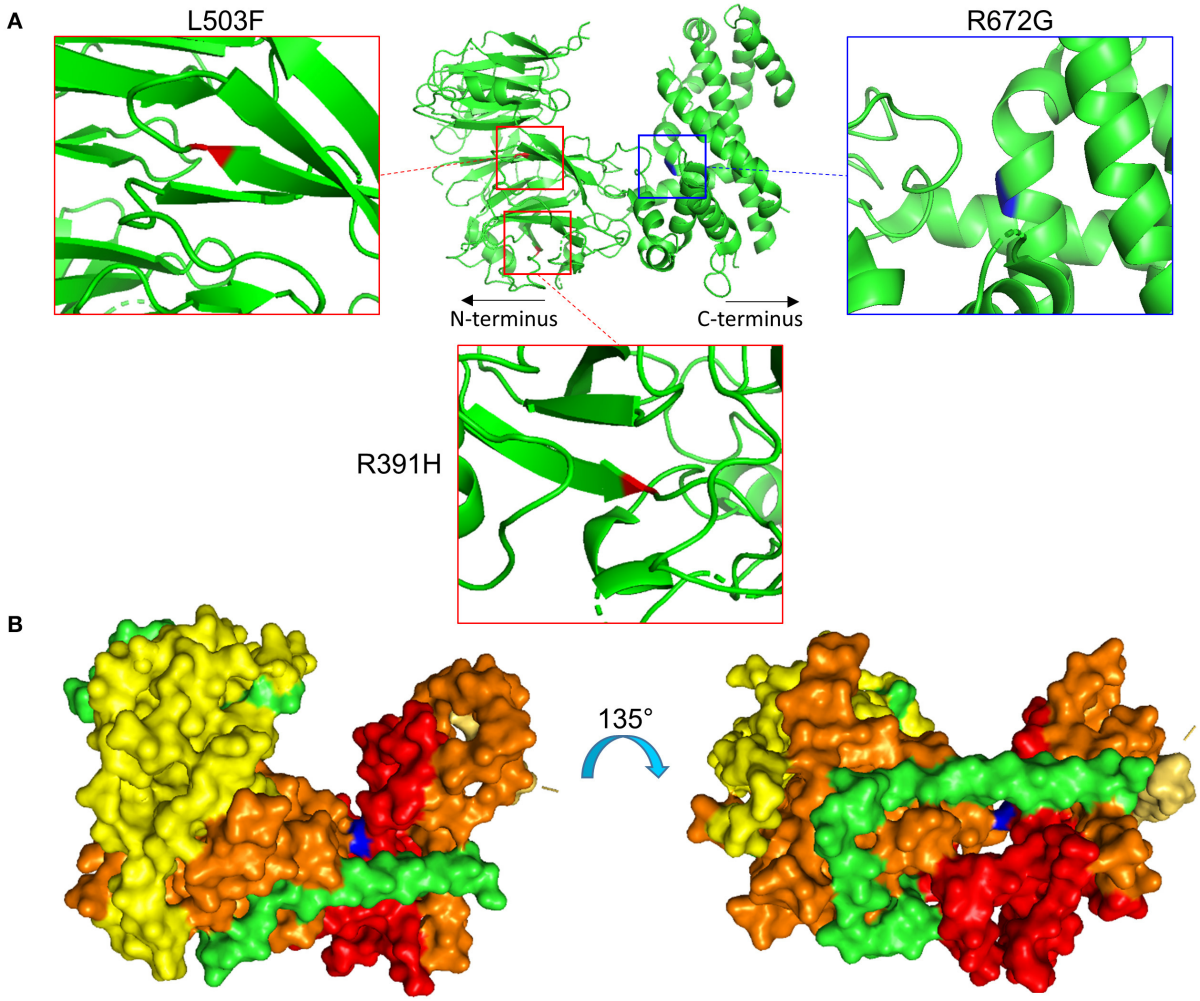
Location of specific *NUP155* variants. **(A)** Predicted 3-dimensional structural distribution of potential pathogenic amino acid substitutions reveal that two clinically associated mutations, R391H and L503F (see text), are found embedded within the beta-propeller feature of NUP155. Our highest prioritized *NUP155* variant, R672G, is found within the alpha-helical region of the crescent shaped domain. Shown is the crescent shaped region, with the two clinical variants highlighted in red and the highest prioritized variant highlighted in blue. Boxes provide magnified views of all three variants. **(B)** Surface rendering of the crescent shaped region of NUP155 reveals central location of the R672G residue (blue) from front and back “sides” of the protein.

**Figure 5:**
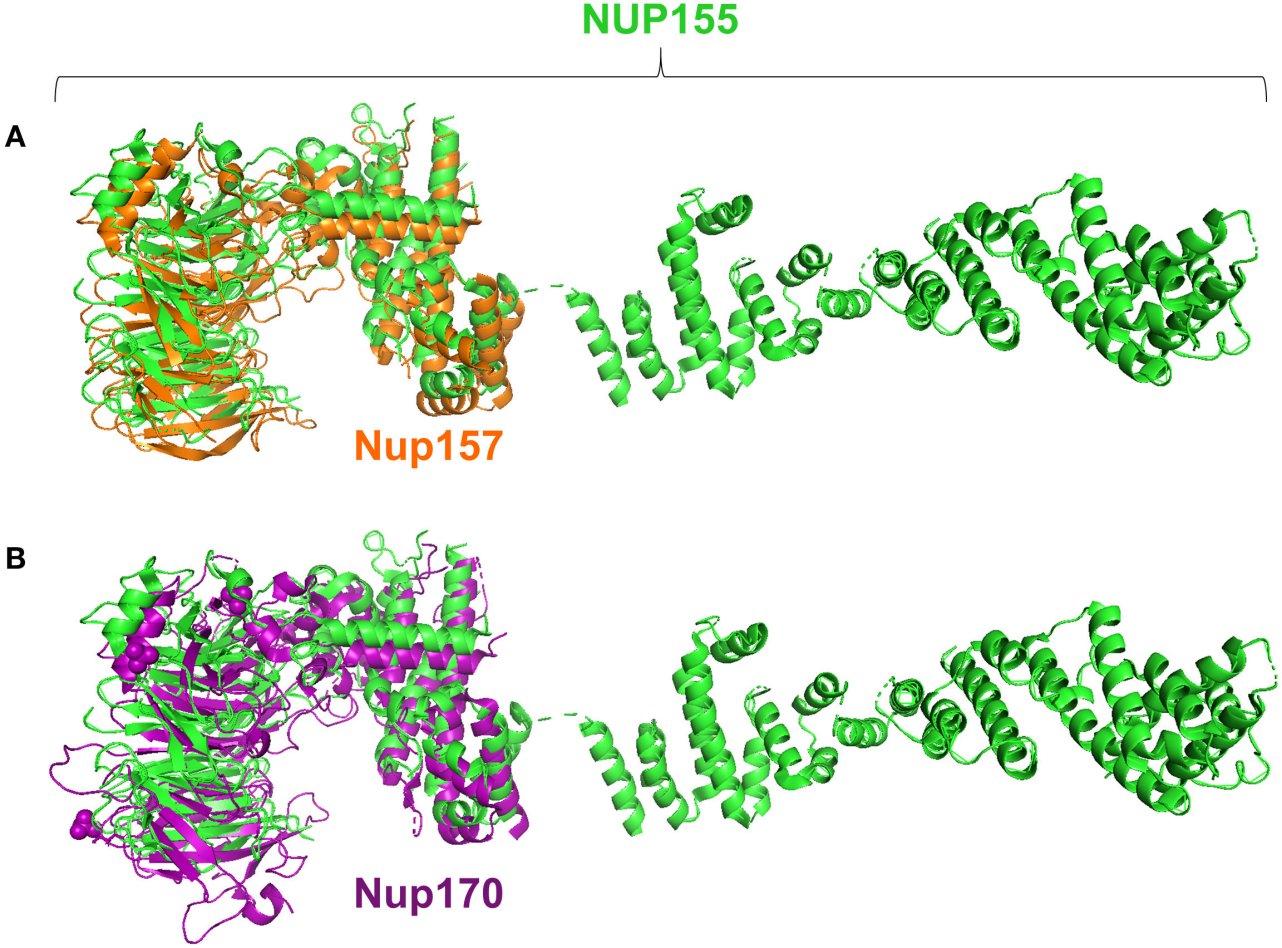
Structure alignments for NUP155 homologs. Analysis revealed that the region in which clustering occurs aligns with the nucleotide binding domains reported for the NUP155 homologs, Nup157 and Nup170 as shown in **(A)** and **(B)**, respectively. Specifically, querying the InterPro database to identify conserved regions of NUP155 identified members of the CATH-Gene3D superfamily 1.20.58.1780 that includes the crystallizable fragments of Nup157 and Nup170. Structure alignments for the homologous fragments with NUP155 reported the closest fit for the nucleotide binding domain of Nup157 with NUP155 (RMSD = 0.280).

Specifically, the atrial fibrillation associated variants R391H and L503F (9, 10, 13) were located within the N-terminal β-propeller domain of this crescent region (Figure 4A). In the present study the majority of predicted damaging variants clustered downstream of the clinically reported alleles R391H and L503F, and were distributed throughout the rest of the C-shaped region and to a lesser extent within the extended C-terminal α-helical stack (Figures 3, 4). The variant coding for R672G (rsID: rs373376199) returned the highest predicted pathogenicity, located within the alpha helical region of the crescent shaped domain (Figure 4A). Surface rendering highlights the R672 residue position within the crescent (Figure 4B). Of note, the crescent shaped region of NUP155 is functionally homologous with nucleotide binding domains for NUP155 (human) homologs Nup157 (fungus) and Nup170 (Yeast, Figure 5) (27, 28).

### NUP155 Protein-Protein Interaction Networks and Topological Analysis

**Figure 6:**
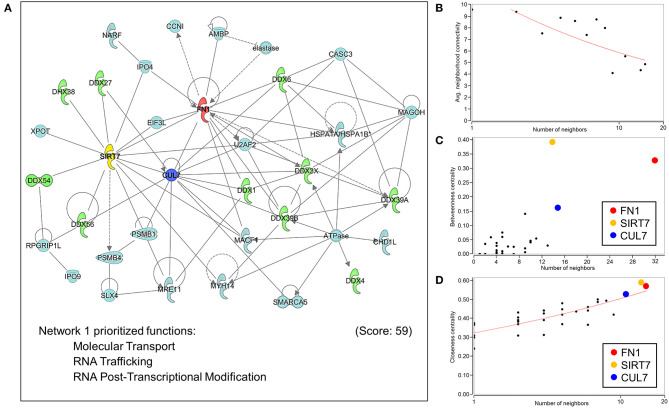
Network analysis of protein-protein interactions of NUP155. NUP155 forms a subnetwork that integrates and enriches specific functional families. **(A)** Network 1 was the highest scoring network comprised of 35 nodes that prioritized Molecular Transport, RNA Trafficking and RNA Post-Transcriptional Modification. Also shown is significant representation of RNA helicases (10 nodes) displayed in green. **(B)** Plotting average neighborhood connectivity against degree/number of neighbors for network 1 revealed disassortative mixing (red line), indicating presence of hubs within the network. **(C,D)** Topology analysis focused on betweenness centrality and closeness centrality revealed the three top hubs as *FN1* (red node), *SIRT7* (yellow node) and *CUL7* (blue node), implicating the role of these three hubs in regulating subnetwork integrity and informational flow.

Extrapolation of inferred human NUP155 protein-protein interactions (PPI) using GeneCards collated data reported a total of 454 potential partners, 441 of which could be mapped to a total of 21 subnetworks in Ingenuity Pathways Analysis. The most significantly enriched molecular and cellular functions for all 441 entities were molecular transport and RNA trafficking. Moreover, 4 out of the top 5 networks prioritize RNA Post-translational Modification and RNA Export/Transport (Supplemental Table 3). The highest scoring network enriched for Molecular Transport, RNA Trafficking, and RNA Post-Transcriptional Modification (Figure 6A, Supplemental Table 3). Topological analysis identified disassortative mixing within this network, with fibronectin 1 (*FN1*), sirtuin 7 (*SIRT7*), and cullin 7 (*CUL7*) emerging as betweenness and closeness centrality hubs (Figures 6B–D).

**Figure 7:**
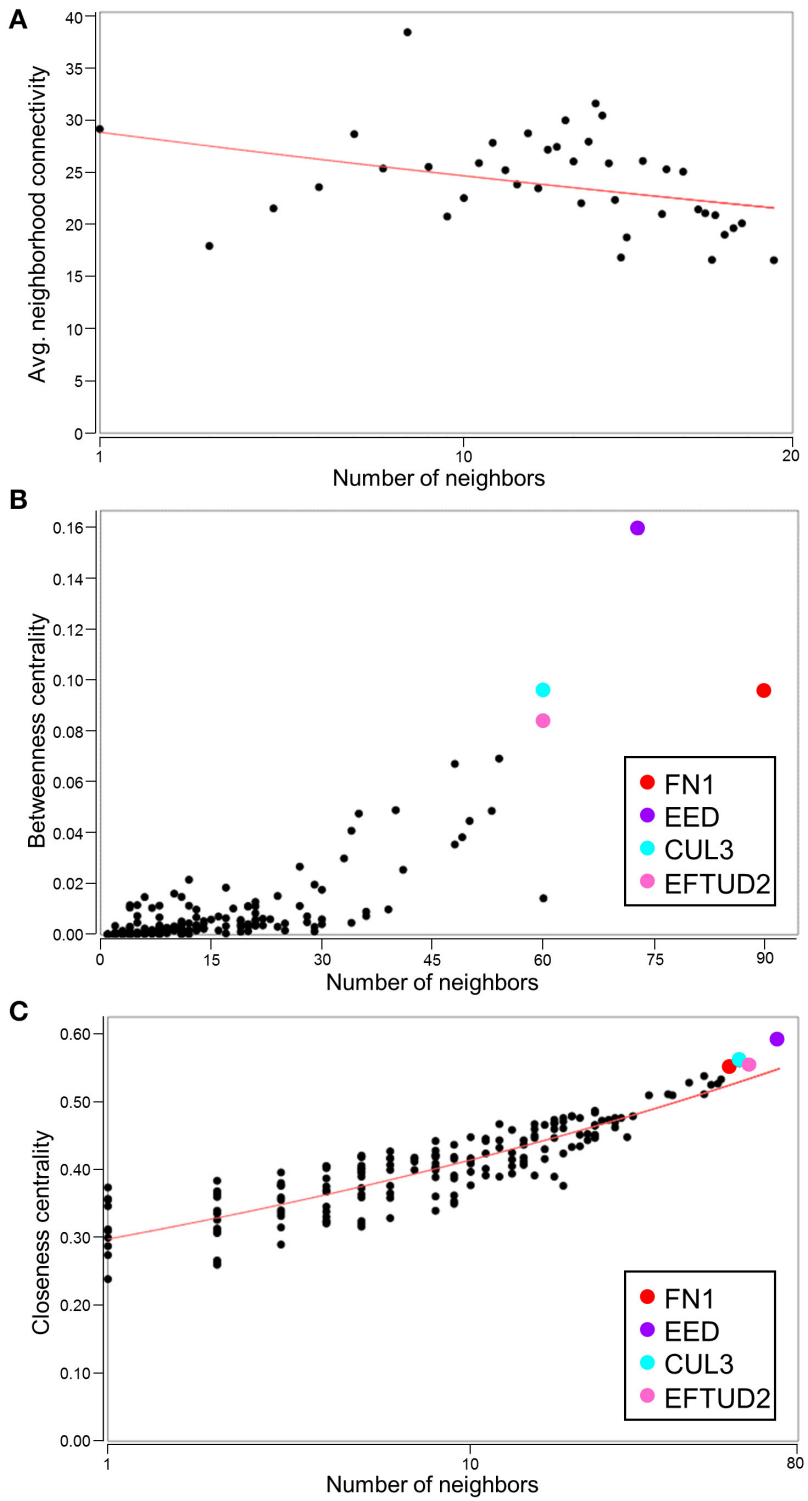
Identification of hubs within the merged network of NUP155 protein-protein interactions. **(A)** Negative slope (red line) indicates that disassortative mixing is preserved in the larger, merged network comprising the top 5 subnetworks (175 nodes), all of which depict RNA trafficking and RNA post-transcriptional modification as highly prioritized functions. **(B,C)** Hubs within the merged network include *FN1* (red), *EED* (purple), *CUL3* (cyan), and *EFTUD2* (pink) in both topological analysis plots of betweenness centrality and closeness centrality.

With the conserved RNA function prioritized in the highest scoring networks, the top 5 subnetworks were merged to investigate hub identities within the larger network. While the disassortative nature of the network was preserved (Figure 7A), several hubs identified by betweenness and closeness centrality analysis differed. High scoring betweenness and closeness centrality nodes included *FN1*, embryonic ectoderm development (*EED*), cullin 3 (*CUL3*) and elongation factor Tu GTP binding domain containing 2 (*EFTUD2*) (Figures 7B,C).

## Discussion

Insights into novel heritable components of cardiopathogenic susceptibility is possible due to the depth of modern high throughput datasets, yet a significant challenge lies in parsing these data to identify pathogenic contributors to disease. In the present study, we used the online gnomAD, dbNSFP, and NHLBI-ESP Exome Variant Server datasets to prioritize NUP155 variants predicted in our study to be potentially pathogenic. These variants clustered within the N-terminal crescent shaped domain of NUP155, and functional enrichment analysis of the inferred protein subnetwork organized by NUP155 returned overrepresentation of multiple RNA regulatory cascades. These included RNA post-translational modification, export, and splicing. This work reports for the first time a bioinformatically driven evaluation of potential NUP155 pathogenicity, and defines functional characteristics and key regulatory hubs associated with the NUP155 subnetwork.

A caveat associated with gnomAD is that individuals with (severe) disease may still be included in data representing the general population. For example, the previously reported atrial fibrillation-associated rare NUP155 variants R391H and L503F had respective allele frequencies of 3.977 × 10^−6^ and 1.193 × 10^−5^ in gnomAD, setting a precedence for the presence of rare cardiopathogenic NUP155 variants in this cohort. Indeed, we identified *NUP155* SNVs with comparable rarities using our predictive algorithm. In general, the presence of these rare alleles, i.e., those with a minor allele frequency (MAF) < 1% may represent non-pathogenic variants as well, as many variants of uncertain significance (VUS) that may be benign allelic variations can occur in large datasets (29, 30). However, in line with our results, secondary validation with online ENSEMBL tools that integrate the robust REVEL, MetaLR and Mutation Assessor metrics (31–33) confirmed prioritization of the NUP155 R672G as a variant of interest in the present study (Supplemental Table 4). Furthermore, all variants in the present study were reported in gnomAD as heterozygous individuals that may represent a carrier background. In such a setting, possibly lethal cardiovascular disease may only manifest in the homozygous condition (10). This may explain the paucity of homozygous allelic distributions for the current prioritized variants, as SNVs resulting in mortality prior to detection may not be reported. In addition, sex-dependent skewing is observed for multiple variants, e.g., V402M, I553M, G754R, D429V, R750H, and R336H (Figure 2) that may indicate a sex-associated predilection for expression of these NUP155 variants. Indeed, gonadal enrichment of specific nup isoforms has been reported (34, 35), however the effects of a non-normal population distribution cannot be ruled out in the present study.

Benchmarking of our method using *SCN5A*, whose gene variants have been shown to be pathogenic for AF (24), revealed that the two pathogenic *SCN5A* variants independently identified in gnomAD, i.e., S216L and F2004L, were detected by our approach but were categorized differently. The rarer S216L variant enriched as disease causing, while the less rare F2004L variant did not. This suggests that our algorithm may be optimized for detecting extremely rare disease causing variants but may miss more common pathogenic ones, and may benefit from implementing robust AF populations as recently demonstrated (36).

The crescent shaped domain within the amino terminal region of NUP155 harbors the clinical R391H and L503F mutations associated with atrial fibrillation. Molecular evolution and conservation analysis indicate that this region is highly conserved within the NPC and is critical for mediating interactions with other inner ring nups (26). Other non-NUP proteins may interact with NUP155. For example, HDAC4 functionally interacts with NUP155 in a neonatal rat ventricular model of cardiac hypertrophy, though this association is mediated by the C-terminal domain of NUP155 with HDAC4 (37). Disruption of these regions prevented functional association of NUP155 and HDAC4, and dysregulates functional chromatin positioning and gene expression. Given that intrinsic autoinhibition of NUP155 is mediated by association of its N- and C- terminal regions (38), the interaction of HDAC4 with the C-terminal domain may affect NUP155 self-inhibition that could result in altered interactions at the N-terminal domain. Alternatively, it is also possible that different protein binding partners associate with discrete regions of NUP155. Of note, although this is speculative at this stage, different missense variants of NUP155 may follow different modes of inheritance, where in some cases heterozygosity is sufficient to impose a clinical phenotype (hence translating into dominant inheritance) while in others a single copy of the variant may cause a sub-clinical effect that becomes overt only in presence of a second copy of the variant (recessive inheritance).

Structural mapping indicates that the amino terminal crescent-shaped domain aligns with the nucleotide binding region identified in NUP155 homologs (27, 28, 39). This was initially proposed and tested by work in the fungal NUP155 homolog Nup157. In that work, a positively charged domain was identified for the crescent shaped region and assays performed with Nup157 fragments confirmed DNA binding activity *in vitro*. This was further validated by independent *in vivo* studies that reported a function for another NUP155 homolog, Nup170p, in regulating subtelomeric chromatin dynamics as well as establishing chromatin tethers that ultimately affected developmental signaling (28). In the present study, this crescent shaped domain harbors a hotspot in which our prioritized variants were enriched, suggesting that NUP155 pathogenicity may be associated with the ability of NUP155 to functionally interact with DNA and/or RNA. It is worth considering that the C-shaped amino terminal portion of NUP155 maintains a defined electrostatic profile (27) that would be sensitive to dramatic changes in local amino acid composition, such as the R672G variant prioritized in the present study. In addition, this region mediates NUP155 interaction with the nuclear envelope membrane and plays a critical role in NPC biogenesis (40) that may impair nucleocytoplasmic transport with effects on the functional transcriptome and/or proteome of the cell. Our previous work in which NUP155 deficiency remodels transcriptome profiles in pluripotent cells (11, 12) supports this, in addition to earlier studies that identified defective import of HSP70 (10) in *nup155* deficient models. Indeed, differential transcriptome/proteome composition could be a significant underlying factor that contributes to impaired cardiogenesis in the presence of preserved NPC assembly and structure, and is an area of future investigation.

Network analysis of predicted NUP155 protein-protein interactions (PPI) revealed significant functional enrichment of a RNA processing and metabolism subnetwork module that indirectly interacts with NUP155. These results are supported by recent analyses of the cardiomyocyte RNA-binding proteome that identified NUP155 as a *bona fide* RNA binding protein (41). In their robust and complementary high throughput proteomic analysis, Liao et al. identified the presence of RNA-binding Rossman fold domains in a subset of proteins within HL-1 cardiomyocytes. Significantly, several nups with direct RNA binding functions, including NUP155, were identified in their analysis. This is in line with earlier work that predicted direct RNA binding functions for NUP155 (27) as well as with the canonical role of NUP155 in mRNA export.

Topological analysis of the NUP155 PPI network revealed several hubs with high betweenness centrality scores. Hubs with these characteristics are essential to maintaining network integrity (12). Of these, *FN1* was identified as a hub with the highest betweenness centrality score and suggests that within an informational signaling context, impacts of NUP155 dysregulation spans nuclear to pericellular microenvironments. This is significant in the context of cardiovascular disease given the well characterized role of fibronectin dysregulation and fibrosis associated with atrial fibrillation (42). The current identified gene network structure suggests that the AF phenotype associated with NUP155 disruption may reflect effects on fibronectin expression dynamics and future work will be necessary to explore this potential functional relationship. The next hub identified in the present analysis is *SIRT7*, an NAD^+^ dependent protein deacetylase and genomic stabilizer that regulates H3K18Ac levels associated with pluripotent replication loci (43). In the context of cardiac development and disease, the sirtuin family, i.e., SIRT1/4/5/6, demonstrate roles in a diversity of processes including energy metabolism, cardiac hypertrophy, heart failure, I/R injury and cardiomyocyte autophagy, while the functions of SIRT7 have specifically been reported to confer protective anti-apoptotic effects on cardiomyocytes by mitigating ROS-induced injury (44). The last of the top 3 hubs identified in the *NUP155* network was *CUL7*, an E3 ubiquitin ligase that promotes mitotic re-entry of cardiomyocytes (45). Thus, *FN1, SIRT7*, and *CUL7* emerge here as hubs that determine integrity and informational flow within the top scoring network of the inferred NUP155 protein-protein interactions. In addition, multiple nodes within the top network were identified as RNA helicases, specifically 1 DEAH (DHX) and 9 DEAD box (DDX) helicases. These enzymes catalyze the unwinding of RNA helices to promote proper conformational dynamics during the synthesis of RNA-protein complexes and structured RNAs (46). Results of the current analysis implicate that disruption of the NUP155 interactome could impact RNA helicase localization, expression and/or activity. In line with this notion is the observation that dysregulation of DDX helicases causes timing delays for a variety of physiological systems including cardiac development (47).

To investigate functional gene ontology enrichment and hub identities within the larger network, the top 5 networks were merged into a collective interactome. Topological analysis revealed that the disassortative mixing observed in the smaller network persisted within the larger framework, although specific hub identities differed. For example, *FN1* maintained its priority as a hub critical for network integrity and informational flow however *EED, CUL3*, and *EFTUD2* were the next most significant hubs with higher betweenness and closeness centrality metrics. The significance of these proteins within the context of CVD has been reported. For example, EED promotes cardiac maturation mediated by interactions of EED with histone deacetylases (48). Similar to CUL7, CUL3 is an E3 ubiquitin ligase that may act as a hierarchical regulator of mammalian cellular differentiation (49). The remaining hub observed in the present study is *EFTUD2*, a U5 small nuclear ribonucleoprotein that forms part of the spliceosomal complex (50) and has been associated with MFDGA syndrome-related congenital heart defects in patients with heterozygous *EFTUD2* loss-of-function mutations (51). Of the genes identified in the present network analysis, only *FN1* has been associated with atrial fibrillation (52). Given the role of these molecules as network hubs however, they may be critical for maintaining integrity of the molecular background to facilitate pathology, as recently demonstrated for RNA binding proteins in the pathogenesis of cardiac fibrosis (53).

The identification of developmental functions for nups, as well as consistent association of discrete nucleoporin mutations with cardiac disease, suggests that this family of proteins may actively contribute to cardiac development and pathology (54–57). Here, we have identified a unique enrichment of NUP155 variants within a hotspot associated with chromatin binding and RNA regulation. In the present analysis, R672G was the most prioritized NUP155 variant out of 24 candidates. Analysis of the predicted NUP155 interactome implicates a variety of binding partners that could be impacted downstream of NUP155 dysfunction, though future functional studies to study these clinical and predicted SNVs in the context of cardiogenesis is necessary. Ultimately, characterization of the systems biology level effects of these NUP155 variants will be critical to understanding and defining a novel determinant of cardiac disease etiology, as well as develop the broader emerging paradigm of nups in development and disease.

## Data Availability Statement

Data for analysis was downloaded from publicly available databases: National Heart Lung Blood Institute-Exome Sequencing Project (NHLBI-ESP) Exome Variant Server (https://evs.gs.washington.edu/EVS/), Genome Aggregation Database (gnomAD; https://gnomad.broadinstitute.org/), and dbNSFP database (https://sites.google.com/site/jpopgen/dbNSFP).

## Author Contributions

RL and CP analyzed data, prepared figures, as well as prepared and edited the manuscript. MG collated NUP155 protein interaction data and provided assistance with written methodologies. YA performed bioinformatic analysis and provided data for figure. AS provided the variant hotspot analysis and figure. RF designed the study, performed bioinformatic analyses, wrote and revised manuscript. All authors have read and approved the manuscript.

### Conflict of Interest

The authors declare that the research was conducted in the absence of any commercial or financial relationships that could be construed as a potential conflict of interest.
