# Corrigendum: Protein Subdomain Enrichment of *NUP155* Variants Identify a Novel Predicted Pathogenic Hotspot

**DOI:** 10.3389/fcvm.2020.630993

**Published:** 2020-12-14

**Authors:** Riley J. Leonard, Claudia C. Preston, Melanie E. Gucwa, Yohannes Afeworki, Arielle S. Selya, Randolph S. Faustino

**Affiliations:** ^1^Genetics and Genomics Group, Sanford Research, Sioux Falls, SD, United States; ^2^Department of Biology, College of St. Benedict/St. John's University, Collegeville, MN, United States; ^3^Department of Biology, Carthage College, Kenosha, WI, United States; ^4^Functional Genomics & Bioinformatics Core Facility, Sanford Research, Sioux Falls, SD, United States; ^5^Behavioral Sciences Group, Sanford Research, Sioux Falls, SD, United States; ^6^Department of Pediatrics, Sanford School of Medicine of the University of South Dakota, Sioux Falls, SD, United States

**Keywords:** single nucleotide variants (SNV), nucleoporins, network biology and protein-protein interactions, atrial fibrillation (AF), nuclear envelope (NE)

In the original article, we neglected to include the funder “National Science Foundation REU grant 1756912 to Melanie E. Gucwa”.

The authors apologize for this error and state that this does not change the scientific conclusions of the article in any way. The original article has been updated.

