# Supplementary material for: Protein Subdomain Enrichment of NUP155 Variants Identify a Novel Predicted Pathogenic Hotspot
Source: Front Cardiovasc Med. 2020 Feb 7;7:8. doi: 10.3389/fcvm.2020.00008 (PMC7019101; doi:10.3389/fcvm.2020.00008)
Supplement: Supplementary file 1 [file Table_1.pdf]

Supplemental Material

Supplemental Table 1. List of 72 *NUP155* variants in our study that were consistently found in dbNSFP, gnomAD and NHLBI-EVS databases, with their respective rsIDs, metrics scores and composite values.

		rs_IDs	Protein Change	PolyPhen2 Score	Grantham Score	PhastCons	GERP	Provean Score	SIFT Score
**Probably damaging**	1	rs373376199	R672G	1	125	1	5.16	-6.5	0.024
	2	rs376271013	P990H	1	77	1	4.8	-1.27	0.017
	3	rs145640004	A1204G	1	60	1	5.11	-3.59	0
	4	rs373119361	R1120Q	1	43	1	5.44	-4.43	0.001
	5	rs202058711	R750H	1	29	1	5.59	-6.5	0
	6	rs376696300	R336H	1	29	1	5.46	-1.99	0.024
	7	rs141688173	D429V	0.999	152	1	5.07	-3.08	0.009
	8	rs142961329	D848H	0.999	81	1	5.85	-6.27	0.001
	9	rs145975462	L866V	0.999	32	1	6.16	-6.5	0.002
	10	rs149244067	L947F	0.999	22	0.995	4.59	-2.61	0
	11	rs370781964	P209L	0.998	98	1	5.83	-5.97	0.007
	12	rs143375056	S371N	0.998	46	1	3.64	-5.97	0.007
	13	rs375239602	P516L	0.997	98	1	5.55	-1.27	0.253
	14	rs145147317	G754R	0.997	125	1	4.49	-6.36	0.002
	15	rs151163391	K1253N	0.996	94	0.971	0.43	-1.46	0.496
	16	rs368777239	F727C	0.995	205	0.999	5.78	-1.91	0.176
	17	rs142350078	S337F	0.994	155	1	5.46	-2.55	0.061
	18	rs376772699	G155D	0.993	94	0.993	4.85	-4.91	0.005
	19	rs148457088	P497L	0.989	98	1	5.41	-4.15	0.112
	20	rs202194194	R1389Q	0.987	43	1	5.05	-5.81	0.141
	21	rs373000659	I553M	0.985	10	0.988	-1.05	-2.83	0.013
	22	rs200783324	G716R	0.984	125	0.998	5.78	-1.65	0.002
	23	rs371676330	P607L	0.975	98	0.985	5.07	-2.16	0.008
	24	rs148814027	V402M	0.971	21	1	5.68	-3.24	0.004
**Possibly damaging**	25	rs367733501	P623L	0.952	98	1	5.07	-2.55	0.013
	26	rs376953622	V1294I	0.941	29	1	4.92	-0.6	0.478
	27	rs143230818	P993L	0.941	98	1	5.71	-5.58	0.003
	28	rs149960589	R786Q	0.919	43	1	5.48	-0.44	0.542
	29	rs148111764	R1096H	0.889	29	1	5.41	-1.55	0.146
	30	rs139891649	R521K	0.884	26	1	5.55	-1.7	0.17
	31	rs139217838	P996T	0.875	38	0.999	5.71	-3.24	0.145
	32	rs376467373	R1076C	0.868	180	0.838	4.81	-2.11	0.206
	33	rs142334950	T448I	0.858	89	0.994	4.2	-3.51	0.004

	34	rs369990299	K939R	0.837	26	0.995	5.46	-1.5	0.332
	35	rs139177869	V658M	0.835	21	1	5.42	-0.83	0.087
	36	rs142078226	R1260H	0.78	29	0.997	5.75	-0.6	0.542
	37	rs373561180	D861G	0.775	94	0.993	6.16	-4.1	0.019
	38	rs371884662	A168V	0.669	64	1	5.72	-1.95	0.017
	39	rs142354357	N664S	0.656	46	1	5.42	-2.34	0.092
	40	rs147213559	N127K	0.642	94	1	4.91	-2.1	0.014
	41	rs373410066	T14A	0.634	58	0.692	1.71	-0.97	0.27
	42	rs148603108	M1289V	0.629	21	1	5.79	-1.7	0.023
	43	rs371603849	R336G	0.621	125	1	5.46	-2.25	0.45
**Benign**	44	rs140076285	S1366C	0.405	112	0.985	5.05	-2.1	0.095
	45	rs372218887	Q1164R	0.387	43	0.994	5.36	-1.28	0.135
	46	rs374698290	T1258A	0.374	58	0.998	5.75	-1.09	0.01
	47	rs138342730	A964S	0.373	99	1	4.42	-2.3	0.038
	48	rs137866662	H542Y	0.27	83	1	4.69	-2.17	0.079
	49	rs140930836	H1328D	0.258	81	1	5.24	-2.57	0.203
	50	rs147865299	S15F	0.214	155	0.637	4.15	-1.18	0.034
	51	rs35326419	G599E	0.138	98	1	5.87		
	52	rs144851445	T1234P	0.087	38	0.376	-3.54	-0.52	0.157
	53	rs139100942	K902T	0.084	78	0.968	4.56		
	54	rs147902256	N435S	0.083	46	1	5.07	0.61	1
	55	rs146473381	S1238L	0.059	145	0.93	5.75	-2.49	0.274
	56	rs375925999	M445V	0.034	21	1	5.07	-1.28	0.008
	57	rs35260031	L1354V	0.03	32	0.996	0.7	-1.17	0.213
	58	rs144607433	V57I	0.027	29	1	5.54	-0.36	0.279
	59	rs367570971	H542Q	0.022	24	1	0.4	-0.94	1
	60	rs368991435	T386M	0.011	81	0.984	3.87	0	0.227
	61	rs61756069	Y614C	0.003	194	0.945	5.07	-1.67	0.082
	62	rs139129279	I156V	0.002	29	0.991	4.56	-0.21	0.536
	63	rs374501700	K1031Q	0.001	53	1	4.35	-0.12	0.646
	64	rs144016077	R883H	0.001	29	0.999	2.05	-1.27	0.136
	65	rs151221761	V827I	0.001	29	1	5.92	0.19	0.881
	66	rs377079846	S414N	0.001	46	1	2.59	-0.43	0.328
	67	rs199530136	N1093S	0	46	0.731	-2.6		
	68	rs372477220	N915S	0	46	0.985	-0.76	0.94	0.938
	69	rs376806446	I805V	0	29	0.965	2.71	0.06	1
	70	rs369621072	I687V	0	29	0.999	2.68	0.18	1
	71	rs111794563	M649T	0	81	1	5.42	-0.17	0.462
	72	rs374935918	M450V	0	21	1	5.52	-0.34	0.166
