# Supplementary material for: Protein Subdomain Enrichment of NUP155 Variants Identify a Novel Predicted Pathogenic Hotspot
Source: Front Cardiovasc Med. 2020 Feb 7;7:8. doi: 10.3389/fcvm.2020.00008 (PMC7019101; doi:10.3389/fcvm.2020.00008)
Supplement: Supplementary file 2 [file Table_2.pdf]

Supplemental Table 2. Population characteristics of potentially cardiopathogenic *NUP155* variants based on gnomAD data.

Variant	Ethnicity							Sex	
	European (non-Finnish)	European (Finnish)	Asian	African	Latino	Ashkenazi Jewish	Other	Male	Female
R672G	0	0	0	2	0	0	0	1	1
R336H	6	0	7	1	0	0	0	2	12
R750H	0	0	0	1	34	0	0	11	24
P990H	1	0	0	0	0	0	0	0	1
R1120Q	1	1	0	0	0	0	0	2	0
A1204G	2	0	1	0	0	0	0	3	0
D429V	150	6	0	1	5	0	4	95	71
D848H	0	0	0	8	1	0	0	3	6
L866V	11	0	0	0	0	0	0	6	5
L947F	1	0	0	0	0	0	0	1	0
P209L	2	0	0	0	0	0	0	2	0
S371N	2	0	0	0	0	0	0	1	1
P516L	4	0	0	0	0	0	0	3	1
G754R	8	0	0	1	0	0	0	8	1
K1253N	0	0	0	1	0	0	0	0	1
F727C	2	0	0	0	0	0	0	0	2
S337F	1	0	1	10	0	0	1	7	6
G155D	1	0	0	0	0	0	0	1	0
P497L	3	0	1	0	0	1	0	4	1
R1389Q	4	0	0	0	3	0	0	4	3
I553M	1	0	0	24	1	0	0	10	16
G716R	8	0	0	1	0	0	1	5	5
P607L	0	0	0	4	0	0	0	1	3
V402M	59	37	85	7	2	2	7	132	67
