# Supplementary material for: Protein Subdomain Enrichment of NUP155 Variants Identify a Novel Predicted Pathogenic Hotspot
Source: Front Cardiovasc Med. 2020 Feb 7;7:8. doi: 10.3389/fcvm.2020.00008 (PMC7019101; doi:10.3389/fcvm.2020.00008)
Supplement: Supplementary file 3 [file Table_3.pdf]

Supplemental Table 3. Display of the most significant functions for the top 5 networks identified within the inferred NUP155 protein-protein interactions.

Network	Score	Top Associated Network Functions
1	59	Molecular Transport, RNA Trafficking, RNA Post-Transcriptional Modification
2	56	Molecular Transport, RNA Trafficking, RNA Post-Translational Modification
3	56	RNA Post-Transcriptional Modification, Molecular Transport, RNA Trafficking
4	53	Cellular Assembly & Organization, Cell-To-Cell Signaling & Interaction, Reproductive System Development & Function
5	50	Cellular Assembly & Organization, RNA Post-Transcriptional Modification, Post-Translational Modification

Note: Functional significance analysis based on IPA Ingenuity Knowledge Base.
