# Supplementary material for: Protein Subdomain Enrichment of NUP155 Variants Identify a Novel Predicted Pathogenic Hotspot
Source: Front Cardiovasc Med. 2020 Feb 7;7:8. doi: 10.3389/fcvm.2020.00008 (PMC7019101; doi:10.3389/fcvm.2020.00008)
Supplement: Supplementary file 4 [file Table_4.pdf]

Supplemental Table 4. Validation of prediction metrics for *NUP155* R672G mutation.

Predictive metrics	Score
SIFT	0*
PolyPhen	1*
REVEL	0.953*
MetaLR	0.952*
Mutation Assessor	0.876*

Note: Comparison of different predictive metrics for R672G mutation (*NUP155* rsID: rs373376199; *Homo sapiens* Transversion G→C) by ENSEMBL including SIFT and Polyphen, two of the metrics used in this study. ENSEMBL predictive metrics included REVEL, MetaLR, and Mutation Assessor. *Scored as pathogenic for respective metric.
